# A simple and fast fabrication of a both self-cleanable and deep-UV antireflective quartz nanostructured surface

**DOI:** 10.1186/1556-276X-7-430

**Published:** 2012-08-01

**Authors:** Jung Suk Kim, Hyun Woo Jeong, Wonbae Lee, Bo Gi Park, Beop Min Kim, Kyu Back Lee

**Affiliations:** 1 Department of Biomedical Engineering, College of Medicine, Korea University, Seoul, 136-701, Korea; 2 Department of Interdisciplinary Bio/Micro System Technology, College of Engineering, Korea University, Seoul, 136-701, Korea; 3 Department of Biomedical Engineering, College of Health Science, Korea University, San 1, Jeongneung-3-dong, Seongbuk-gu, Seoul, 136-703, Korea; 4 Research Institute of Health Science, Korea University, San 1, Jeongneung-3-dong, Seongbuk-gu, Seoul, 136-703, Republic of Korea

**Keywords:** Antireflection, Superhydrophobicity, Nanostructure, Mask-free, Deep-UV

## Abstract

Both self-cleanability and antireflectivity were achieved on quartz surfaces by forming heptadecafluoro-1,1,2,2-tetrahydrodecyltrichlorosilane self-assembled monolayer after fabrication of nanostructures with a mask-free method. By exposing polymethylmethacrylate spin-coated quartz plates to O_2_ reactive ion etching (RIE) and CF_4_ RIE successively, three well-defined types of nanopillar arrays were generated: A2, A8, and A11 patterns with average pillar widths of 33 ± 4 nm, 55 ± 5 nm, and 73 ± 14 nm, respectively, were formed. All the fabrication processes including the final cleaning can be finished within 4 h. All nanostructured quartz surfaces exhibited contact angles higher than 155° with minimal water droplet adhesiveness and enhanced transparency (due to antireflectivity) over a broad spectral range from 350 to 900 nm. Furthermore, A2 pattern showed an enhanced antireflective effect that extends to the deep-UV range near 190 nm, which is a drawback region in conventional thin-film-coating approaches as a result of thermal damage. Because, by changing the conditions of successive RIE, the geometrical configurations of nanostructure arrays can be easily modified to meet specific needs, the newly developed fabrication method is expected to be applied in various optic and opto-electrical areas.

**PACS codes:** 06.60.Ei; 81.65.Cf; 81.40.Vw.

## Background

Numerous studies of surface nanostructures have been conducted to investigate enhancement of the properties of bulk materials to improve their selectivity, applicability, and effectiveness. During the past decades, the technological basis for nanofabrication has been developed by vigorous efforts to develop next-generation lithography for highly resolved patterns up to the industrial level of semiconductor production. Nanofabrication techniques using transparent materials such as quartz comprise one of the most attractive approaches to optical and opto-electrical studies as well as to highly sensitive biosensor fields since quartz is commonly employed in these fields [1-4].

Various methods are used to achieve antireflective property in quartz or glass to increase light transmission. Single- or multilayered thin film coatings, porous coatings, and fabrication of sub-wavelength nanostructures on surfaces using conventional lithography have been the focus of many studies [5-7]. However, the aforementioned conventional methods have some drawbacks. For example, it is difficult to maintain long-term stability of multilayer polymeric coatings because multilayers are unstable in humid environments and temperature changes, and most polymeric materials have strong absorption in the UV region [8]. Moreover, single- or multilayer fabrication methods are only effective for a narrow spectral range. Lithography techniques have the shortcomings of being time consuming, expensive, and restricted to small areas.

In comparison with thin-film-coated surfaces, directly patterned surfaces usually guarantee good mechanical stability because they are free from adhesion problems and tensile stress. Recently, a few techniques for the direct fabrication of sub-wavelength nanostructures on quartz or glass surfaces have been attempted by several groups. Lohmüller et al. and Christopher et al. used block copolymer micelle lithography with reactive ion etching (RIE) and reported that array pattern of a quartz nanostructure showed excellent antireflectivity and anti-fogging in UV and deep-UV region [1,9]. Li et al. applied nanosphere lithography using PS microspheres 210 nm in diameter and reported broad spectrum antireflectivity from 300 to 800 nm and anti-fogging [10]. If a simpler and faster technique is available to obtain appropriate nanostructures, it is highly desirable. Hein et al. reported an innovatively simple and fast method of nanostructure fabrication on glass surfaces by performing RIE after deposition of an approximately 10-nm-thin lithographically unstructured metallic layer onto the surface [11]. We also reported a simple and fast mask-free approach to fabricate nanostructures, which uses two-step RIE (O_2_ and CF_4_ RIE) of polymer-coated quartz in our previous studies [3,4].

Superhydrophobic behavior of a surface can also be achieved by introducing micro- and/or nanostructures at the surface. A superhydrophobic surface is usually defined as having a contact angle greater than 150°. The self-cleaning effect refers to cases in which contaminant particles adhered to a surface are easily washed off with rolling of water droplets. To have a self-cleaning effect, the surface should possess minimized adhesion properties as well as superhydrophobicity [12-14].

The mimetic fabrication of a superhydrophobic surface was primarily inspired by the self-cleanable leaves of the lotus, which have an array of protrusions on the surface [15,16]. The fabrication of self-cleanable surfaces has received a great deal of attention in various novel applications, such as for easy removal of undesirable contaminants from the surface of semiconductors and solar cells, prevention of water corrosion on the exterior skins of automobiles and building units, biomaterials used in clinical therapies that require minimal contamination, no-mass-loss transport of water droplet systems, and microarrays that require specific wetting properties of the substrates for precise spotting [17]. Especially in dry condition, which is common in optical or opto-electric applications, the superhydrophobic surface is expected to decrease the adhesion of dusts because the surface energy in surface-air interface decreases as the surface becomes superhydrophobic.

We previously reported a systematic approach to obtain a superhydrophobic surface with tunable adhesiveness and suggested a useful nanofabrication strategy for achieving self-cleanability that involved fabrication of pillar arrays without dead-end nanopores covered with low-surface-energy materials [12,14]. In this study, we demonstrate both the remarkable broad spectrum antireflectivity including deep-UV region and self-cleanability in nanostructured quartz surfaces using our mask-free fabrication method.

## Methods

The nanopillar arrays with various pillar diameters and inter-pillar distances were fabricated on quartz plates by a mask-free approach. A quartz wafer plate (Buysemi, Suwon, Gyeonggi-do, Korea) was cleaned with piranha solution and then rinsed thoroughly with deionized (DI) water. After heating at 100°C for about 5 min, 950 PMMA A2, 495 PMMA A8, and 950 PMMA A11 (MicroChem Corp., Newton, MA, USA) were each spin-coated onto the surface at 4,000 rpm for 25 s. The expected thicknesses of the PMMA layers reported in the technical support information of the MicroChem products are 50, 500, and 800 nm for A2, A8, and A11, respectively.

Post-baking was conducted for 30 min at 170°C. The PMMA-coated quartz plates were then subjected to reactive ion etching in O_2_ plasma for 1 min and CF_4_ plasma for 10 min at 250 W of RF power, 40 mTorr, and 40 sccm using a custom-made RIE system. To remove the organic remnants from the quartz pattern, each pattern was sintered at 1,000°C in a furnace for 1 h, cleaned with piranha solution, rinsed with DI water, and dried. The surface of each pattern was covered with a self-assembled heptadecafluoro-1,1,2,2-tetrahydrodecyltrichlorosilane (HDFS; Gelest Inc., Morrisville, PA, USA) monolayer using a 3-mM solution of HDFS in n-hexane to reduce the surface energy. All the fabrication processes including the final cleaning and self-assembled monolayer formation could be finished within 4 h.

The morphological images of the nanopillars were obtained by field emission scanning electron microscopy (FESEM; Jeol JSM6710F, Jeol Ltd., Tokyo, Japan). The surface-wetting properties were evaluated by an Easydrop goniometer (KRÜSS, Hamburg, Germany), and the dynamic angle was evaluated using a DSA 100 goniometer (KRÜSS). The advancing and receding contact angles were obtained from more than three points in each specimen by the sessile drop technique. To investigate optical performance, we measured the transmission and reflection properties of the nanostructured quartz plates using a UV-visible spectrometer (Optizen 3220UV, Mecasys Co., Ltd., Daejeon, Korea).

## Results and discussion

**Figure 1:**
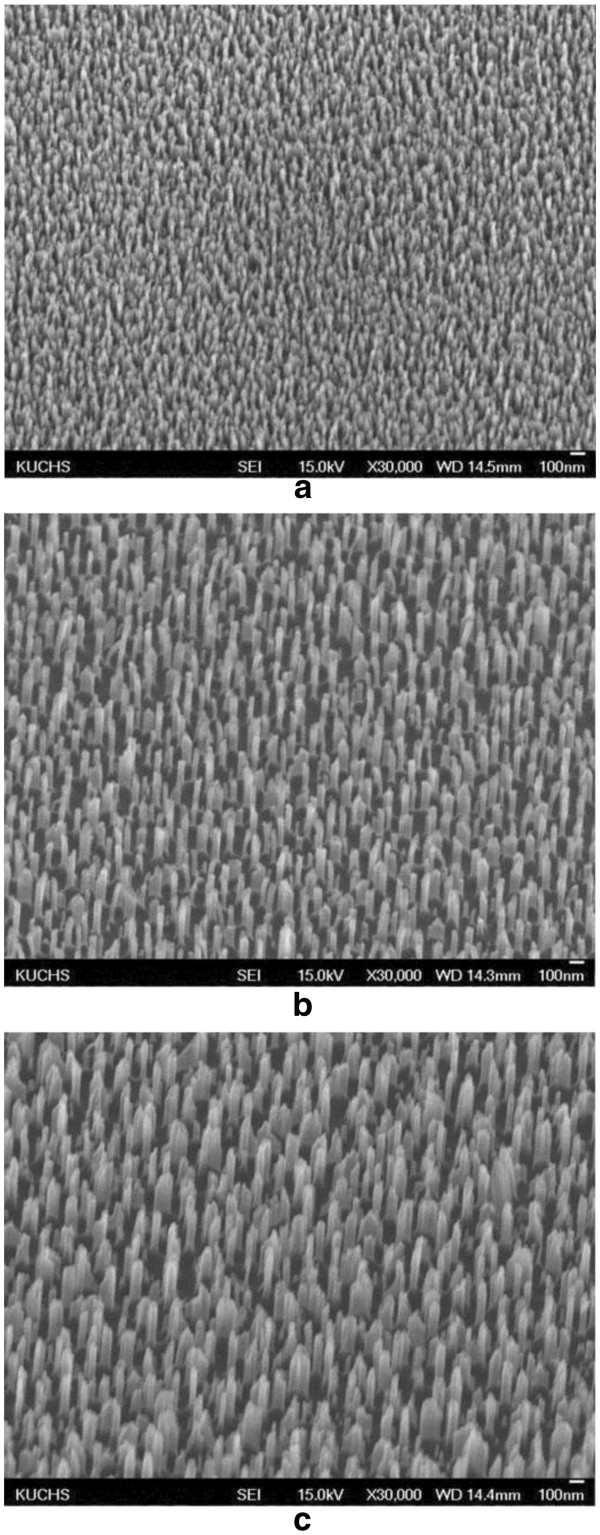
**SEM images of nanopillar array manufactured by new mask-free lithography with various PMMA resists. (a)** PMMA A2, **(b)** PMMA A8, and **(c)** PMMA A11.

Figure 1 shows oblique FESEM images of the fabricated nanopillar arrays obtained for each PMMA resist using the mask-free method. The widths of the quartz nanopillars with A2, A8, and A11 PMMA resists were estimated to be 33 ± 4, 55 ± 5, and 73 ± 14 nm, respectively, from the FESEM images. The heights of the pillars were 95 ± 10, 200 ± 15, and 265 ± 15 nm, respectively. The average pillar diameters, pillar heights, and inter-pillar distances were uniform all over the area (2.5 × 2.5 cm^2^) in each sample. These results demonstrate that it is feasible to systematically control the dimensional features of the pillar pattern. Specifically, larger and higher pillars can be formed by controlling the thickness of the PMMA resist. The mechanism of the nanostructure formation was previously reported [4]. Explaining briefly, pillar-like nanostructures can be fabricated by the O_2_ and CF_4_ two-step RIE process because, by controlling the RIE conditions appropriately, CF_4_-resistant C_x_F_y_ polymeric mask is automatically and selectively deposited during the CF_4_ RIE process on the top of the dot-like nanostructures of the PMMA resist, which are formed during the preceding O_2_ RIE process.

**Figure 2:**
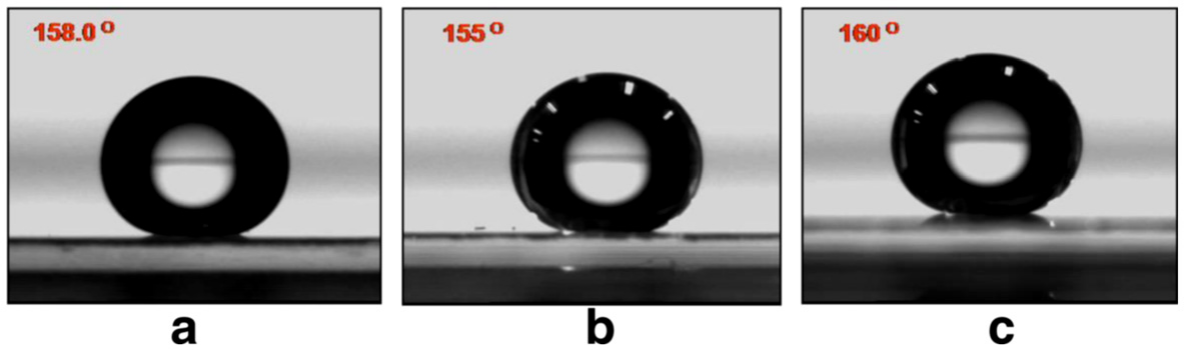
**Contact angle measurements of the structured quartz surface.** Prepared using **(a)** PMMA A2, **(b)** PMMA A8, and **(c)** PMMA A11.

Table 1 shows the static, advancing, and receding water contact angles of the HDFS-modified nanopillar quartz patterns. Photographs of the static water droplets on each patterned surface are also shown in Figure 2. The static contact angles were measured at more than three points in each specimen, and the average values were acquired. A quartz surface is known to be hydrophilic due to the existence of hydroxyl groups on the surface, which is the reason why a quartz surface is easily contaminated by dusts, because nature wants to decrease interfacial energy between the hydrophilic quartz and the most hydrophobic air. In order to make the quartz surface hydrophobic, so as to decrease the interfacial energy, a covalently immobilized monolayer of HDFS molecules was formed on its surface, and the hydrophobicity could be further increased to superhydrophobicity by the nanostructures. All of the HDFS-treated nanopatterns had dynamic contact angles (Table 1) in the superhydrophobic range (greater than 150°) with low hysteresis of about 10°, which demonstrates the self-cleaning effect (Additional file 1: Video S1). In contrast, the HDFS-modified surface of a plain quartz has an advancing angle of about 120° with large hysteresis (40.0°).

**Table 1:** Contact angles of the nanostructured quartz

	**Plain quartz**	**A2 pattern**	**A8 pattern**	**A11 pattern**
*θ*_adv_ (°)	119.0 ± 0.9	158.0 ± 1.4	157.4 ± 1.4	159.0 ± 1.0
*θ*_rec_ (°)	79.0 ± 2.5	145.0 ± 7.0	148.2 ± 3.8	147.0 ± 2.0
Hysteresis	40.0	13.0	9.2	12.0

*θ*_adv_, advancing water contact angle; *θ*_rec_, receding water contact angle.

In nature, some insects have sub-wavelength scale structure patterns with nipple-like or tapered profiles on the cornea that exhibit a gradient in the refractive index between the air and tissue interface. These characteristics play an important role in increasing light transmission. Theoretically, for a thin-film coating, overall reflectance can be a function of antireflection (AR) layer thickness *d* and the wavelength *λ*. For a graded-index transition, substantial antireflection can be obtained when the ratio *d*/*λ* is about 0.4 or higher [1,10]. To enhance the transmission of light and suppress the reflection, the structural size has to be in the sub-wavelength range. In the spectral region from UV to visible light, the structural dimension has to be smaller than 200 nm [1,9].

**Figure 3:**
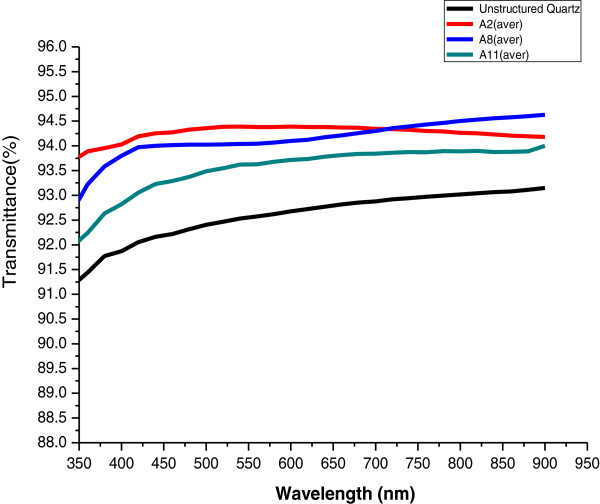
**Transmission properties of structured quartz as determined by UV-visible spectrometry.** Prepared using PMMA A2, PMMA A8, and PMMA A11 with unstructured quartz as a reference.

**Figure 4:**
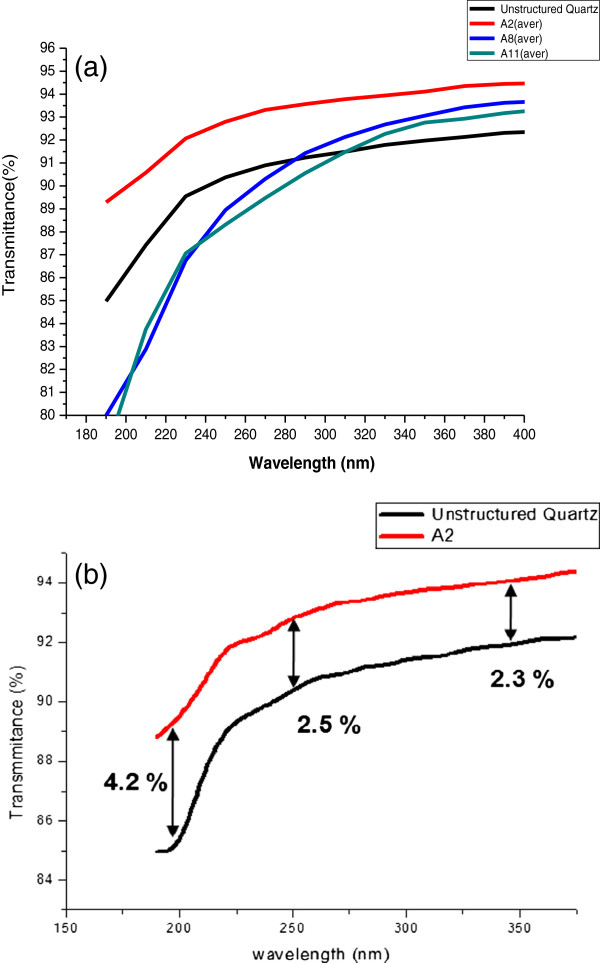
**Transmission properties of structured quartz in the deep-UV region.** Prepared using **(a)** PMMA A2, PMMA A8, PMMA A11, and unstructured quartz; **(b)** high antireflective performance of structured quartz prepared using PMMA A2.

Figure 3 shows the transmission properties of the structured quartz manufactured using A2, A8, and A11 PMMA resists. Transmission data from unstructured quartz samples were used as a reference. All structured quartz prepared using A2, A8, and A11 showed improved transmission of about 2% to 3% over unstructured quartz in a broad spectral range from the UV to the infrared (IR) region (350 to 900 nm). The structured quartz manufactured using an A2 resist demonstrated transmission superior to the unstructured quartz even in the deep-UV range from 190 to 300 nm (Figure 4). This deep-UV range is usually not covered by the conventional polymer AR-coating method. The antireflective property of the structured quartz using A2 varies from approximately 4.2% improvement at around 193 nm to 2.3% at around 340 nm, as indicated by the black arrow. The transmission of the structured surface using A8 and A11 is lower than that of the unstructured quartz below 300 nm, and this is partly the result of light scattering introduced during the fabrication process. In the region from the visible (350 nm) to IR (900 nm) range, the nanostructured quartz prepared using A8 exhibited a stable and uniform antireflective effect and a better optical performance above the 700-nm region than that obtained using the quartz prepared using A2. These experimental results are in good agreement with the aforementioned theories related to the height of nanopillars.

## Conclusions

**Figure 5:**
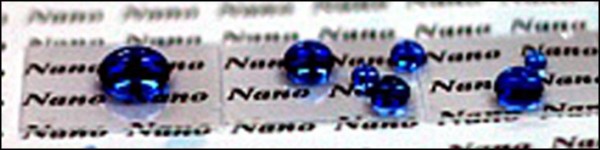
Water droplets on a large surface of the superhydrophobic quartz.

The mask-free method presented here may have several advantages. First, long-term stability is expected because of the superhydrophobic self-cleaning effect. Second, although the area can be restricted by the stage size of a RIE device, a large area can be achieved because our method requires no masks. Figure 5 shows a large patterned area of 3.0 × 3.0 cm^2^. Third, our technology can be employed for industrial optical devices, optical components, and interior and exterior materials requiring both self-cleanable and antireflective properties. In addition, the manufacturing cost is minimal since the fabrication process is simple and fast, and requires no mask.

In the near future, after proper optimization, we plan to apply our technique to other optical components, such as lenses and optical filters, to demonstrate the wide applicability of our technology. Furthermore, we will determine whether the antireflective spectral regions can be controlled using different PMMA resists by changing the parameters of the surface structures such as height, width, and pitch.

## Competing interests

The authors declare that they have no competing interests.

## Authors' contributions

JSK prepared the samples, evaluated surface characteristics, and drafted the manuscript. HWJ evaluated the optical properties, analyzed the data, and drafted the manuscript. WL participated in the design of the study and drafted the manuscript. BGP evaluated the surface characteristics of the samples. BMK and KBL conceived of the study together and participated in its design and coordination. All authors read and approved the final manuscript.

## Supplementary Material

Additional file 1 Video S1. Video shows the rolling of water droplets on the self-cleanable nanostructured quartz surface prepared using PMMA A2, PMMA A8, and PMMA A11. Click here for file
